# AIP1-mediated actin disassembly is required for postnatal germ cell migration and spermatogonial stem cell niche establishment

**DOI:** 10.1038/cddis.2015.182

**Published:** 2015-07-16

**Authors:** J Xu, P Wan, M Wang, J Zhang, X Gao, B Hu, J Han, L Chen, K Sun, J Wu, X Wu, X Huang, J Chen

**Affiliations:** 1 State Key Laboratory of Pharmaceutical Biotechnology and MOE Key Laboratory of Model Animals for Disease Study, Model Animal Research Center, Nanjing University, Nanjing, China; 2 State Key Laboratory of Reproductive Medicine, Nanjing Medical University, Nanjing, China; 3 Bio-X Institute, Shanghai Jiaotong University, Shanghai, China

## Abstract

In mammals, spermatogonial stem cells (SSCs) arise from early germ cells called gonocytes, which are derived from primordial germ cells during embryogenesis and remain quiescent until birth. After birth, these germ cells migrate from the center of testicular cord, through Sertoli cells, and toward the basement membrane to form the SSC pool and establish the SSC niche architecture. However, molecular mechanisms underlying germ cell migration and niche establishment are largely unknown. Here, we show that the actin disassembly factor actin interacting protein 1 (AIP1) is required in both germ cells and Sertoli cells to regulate this process. Germ cell-specific or Sertoli cell-specific deletion of *Aip1* gene each led to significant defects in germ cell migration after postnatal day 4 or 5, accompanied by elevated levels of actin filaments (F-actin) in the affected cells. Furthermore, our data demonstrated that interaction between germ cells and Sertoli cells, likely through E-cadherin-mediated cell adhesion, is critical for germ cells' migration toward the basement membrane. At last, *Aip1* deletion in Sertoli cells decreased SSC self-renewal, increased spermatogonial differentiation, but did not affect the expression and secretion levels of growth factors, suggesting that the disruption of SSC function results from architectural changes in the postnatal niche.

In mammals, spermatogenesis and male fertility depend on the self-renewing and differentiating functions of spermatogonial stem cells (SSCs), which are regulated by cues from the niche microenvironment.^1^ During embryogenesis, the precursors of SSCs can be traced to primordial germ cells (PGCs) in the proximal epiblast at embryonic day 6.25 (E6.25), which migrate to genital ridge and together with somatic cells there to form the embryonic gonad.^2^ The PGCs then differentiate to gonocytes (also called prespermatogonia), proliferate for a brief period of time, and then remain mitotically quiescent until birth.^3, 4, 5^ After birth, these neonatal germ cells (gonocytes) located at the center of testicular cord become proliferative and relocate themselves from the center toward the basement membrane of each testicular cord.^4, 6^ During the migration or relocation process, germ cells associate with and move through the Sertoli cells, the sole somatic cell type within the testicular cord and the major component of the SSC niche. After reaching the basement membrane at the periphery, most of these germ cells adopt a distinct morphology and become the undifferentiated spermatogonial population, which includes SSCs and other non-stem cell progenitors,^7, 8, 9^ supposedly in response to cues from the supporting cells. It has been suggested that the postnatal germ cell migration is crucial for the formation of SSC pool and the establishment of the SSC niche architecture. However, the mechanisms underlying these two processes are not well understood.

In neonatal mice, germ cells specifically express the cell adhesion molecule E-cadherin on the cell surface,^10, 11^ whereas other adhesion markers including N-cadherin and *β*1-integrin were found in both germ cells and Sertoli cells.^12, 13, 14^ However, whether these adhesion molecules have specific roles in germ cells' outward migration and subsequent differentiation were not yet known. In *Drosophila* testis, the germline stem cells (GSCs) were shown to attach to the somatic hub cells (a major niche component) via membrane bound E-cadherin in both cell groups, and disruption of E-cadherin-mediated cell adhesion between GSCs and hub cells severely affected self-renewal and maintenance of GSCs.^15, 16^ Moreover, a recent study showed that the actin polymerization regulator profilin is required to localize and maintain E-cadherin to the GSC-hub cell interface and is thus essential for the maintenance of GSCs. This result is consistent with findings in other systems that dynamics of actin cytoskeleton directly regulate the assembly and maintenance of E-cadherin-based cell adhesion.^17^ Interestingly, we have previously shown that actin interacting protein 1 (AIP1), an actin disassembly factor, regulates E-cadherin distribution and dynamics during a cell rearrangement process of the *Drosophila* eye disc.^18^ AIP1 has been shown to act together with cofilin/actin-depolymerizing factors to promote actin dynamics in various cellular processes, and it is highly conserved in all eukaryotes examined so far.^19, 20, 21, 22, 23, 24^ Here, we utilized germ cell- or Sertoli cell-specific deletion of *Aip1* (also known as *Wdr1*) in the murine testis to study the process of germ cell migration and SSC niche establishment.

## Results

### *Aip1* deletion in Sertoli cells or germ cells caused severe defects in spermatogenesis

**Figure 1:**
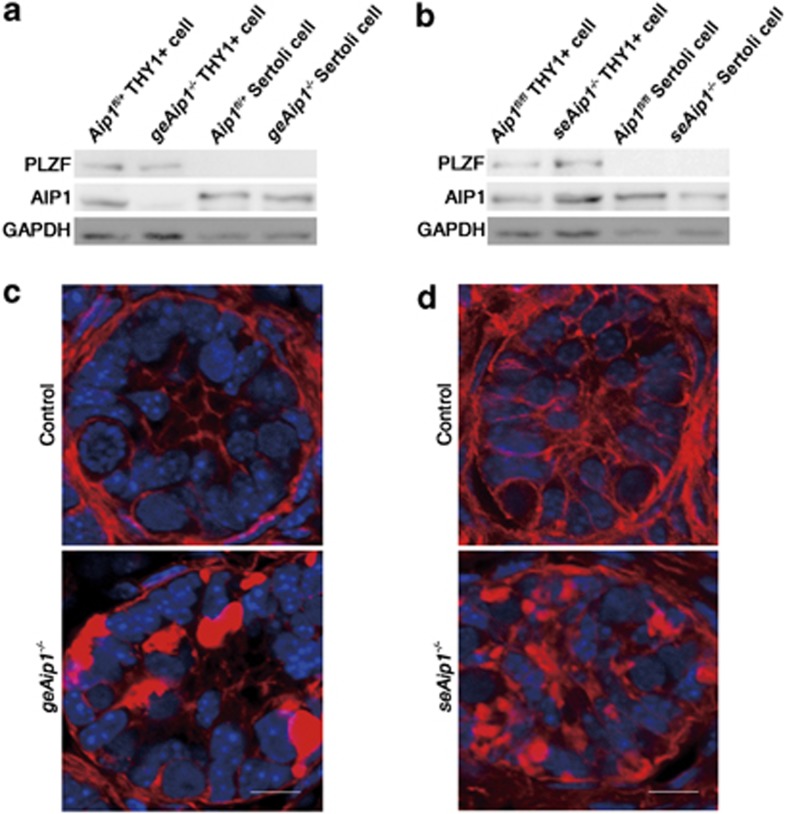
Reduction of AIP1 level and increase of F-actin level as the results of *Aip1* deletion in either Sertoli cells or germ cells. (**a** and **b**) Western blot analysis of AIP1 protein levels in isolated THY1+ germ cells from *geAip1*^−/−^ testes at P7 (**a**) and isolated Sertoli cells from *seAip1*^−/−^ testes at P7 (**b**). The control for (**a**) is THY1+ germ cells from *Aip1*^*fl/+*^ testes and it is compared with THY1+ germ cells from *geAip1*^−/−^. The control for (**b**) is Sertoli cells from *Aip1*^*fl/fl*^ testes and it is compared with Sertoli cells from *seAip1*^−/−^ (**c** and **d**) Representative confocal images of phalloidin staining (red) of tissue sections from the P7 controls, *geAip1*^−/−^ (**c**) and *seAip1*^−/−^ (**d**) testes. Cell nuclei were stained with DAPI (blue). Scale bars: 10 *μ*m

First, we utilized the *Aip1* (*Wdr1*) conditional knockout mouse model (*Aip1*^*fl/fl*^), which contained *lox*P sites flanking the exon 4 of the gene, leading to the premature termination of *Aip1* translation (details of the conditional knockout construct has been reported by Yuan *et al.*^25^). To achieve deletion of *Aip1* specifically in early developing testis, we crossed the *Aip1*^*fl/fl*^ with *Amh* (anti-müllerian hormone)-cre mice that express the cre recombinase in Sertoli cells starting from embryonic day 15 (E15).^26, 27^ To obtain germ cell-specific *Aip1* knockout, we crossed *Aip1*^*fl/fl*^ with *Vasa-cre* mice that express cre in the germline beginning from E15.^28^ Western blot analysis (with a previously referenced anti-AIP1 antibody.^25^) of THY1+ germ cells extracted from testes of postnatal day 7 (P7) *Aip1*^*fl/*^^−^*:Vasa*^*+/cre*^ (referred to as *geAip1*^−/−^ subsequently) mice showed an almost complete removal of the AIP1 protein, indicating an efficient deletion of *Aip1* in the germ cells (Figure 1a). Western blot of Sertoli cells from testes of P7 *Aip1*^*fl/fl*^*:Amh*^*+/cre*^ (s*eAip1*^−/−^) mice exhibited 60% reduction of AIP1 protein level, indicating a partial loss-of-function of *Aip1* in the Sertoli cells (Figure 1b). Finally, the marked increase of F-actin levels, as shown by phalloidin staining, in the P7 *geAip1*^−/−^ germ cells (Figure 1c) and P7 *seAip1*^−/−^ Sertoli cells (Figure 1d), are highly consistent with our previous characterization of *Aip1*'s loss-of-function actin phenotypes in *Drosophila* and mouse.^18, 25^

**Figure 2:**
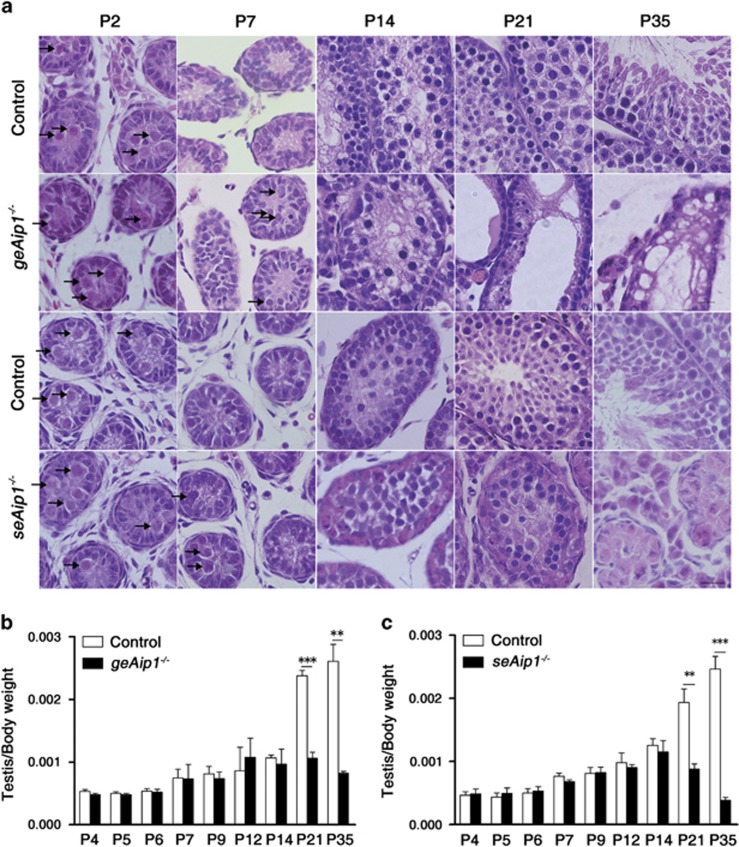
Severe spermatogenesis defects in *geAip1*^−/−^ and *seAip1*^−/−^ mice. (**a**) HE staining of tissue sections of testes from the controls, *geAip1*^−/−^ and *seAip1*^−/−^ mice at the indicated postnatal dates. No significant morphological defects can be seen in *geAip1*^−/−^ and *seAip1*^−/−^ testes at P2. At P7, germ cells in both *geAip1*^−/−^ and *seAip1*^−/−^ testes displayed abnormal positioning of a significant portion of germ cells in the central region of testicular cord, whereas almost all the germ cells in the control testes at P7 would have their migration and positioned themselves adjacent to the basement membrane. At P14, *geAip1*^−/−^ and *seAip1*^−/−^ testes displayed distinct phenotype. *geAip1*^−/−^ testes at P14 displayed apparent reduction in the number of spermatocytes, whereas *seAip1*^−/−^ testes showed no significant reduction in the number of spermatocytes as compared with the control. At P21 and P35, in which round spermatids and spermatozoa begin to form, respectively, *geAip1*^−/−^ testes exhibited marked reduction in the number of spermatids (at P21) and spermatozoa (at P35), indicating that spermatogenesis were effectively blocked at this stage. Similarly, *seAip1*^−/−^ testes also displayed severe reduction of spermatids and spermatozoa. And both *geAip1*^−/−^ and *seAip1*^−/−^ males are sterile. Arrows point to germ cells that were positioned in the central region of the testicular cord and had not reached the basement membrane. Scale bars: 10 *μ*m. (**b** and **c**) Ratios of testis/body weight were significantly reduced in the P21 and P35 *geAip1*^−/−^ and *seAip1*^−/−^ mice as compared with the controls. The strong size reduction in both *geAip1*^−/−^ and *seAip1*^−/−^ testes at P21 and P35 are consistent with the severe defects of spermatogenesis occurring in both knockout mice. Data are presented as means±S.E.M.; *n*≥3 in each group; **P<0.01, ***P<0.001

Hematoxylin and eosin (HE) staining revealed that the earliest observable defects occurred at P7 when a significant portion of germ cells in both *geAip1*^−/−^ and *seAip1*^−/−^ testes failed to relocate to the basement membrane (Figure 2a). Further HE and immunofluorescence analyses from P12 to P35 showed that *Aip1* ablation in the germ cells and Sertoli cells both caused severe defects in later stages of spermatogenesis and *Aip1* was likely required for the later proliferation and meiosis of germ cells (Figure 2 and Supplementary Figure 1).

### *Aip1* is required in both Sertoli cells and germ cells for the outward migration of germ cells to basement membrane

**Figure 3:**
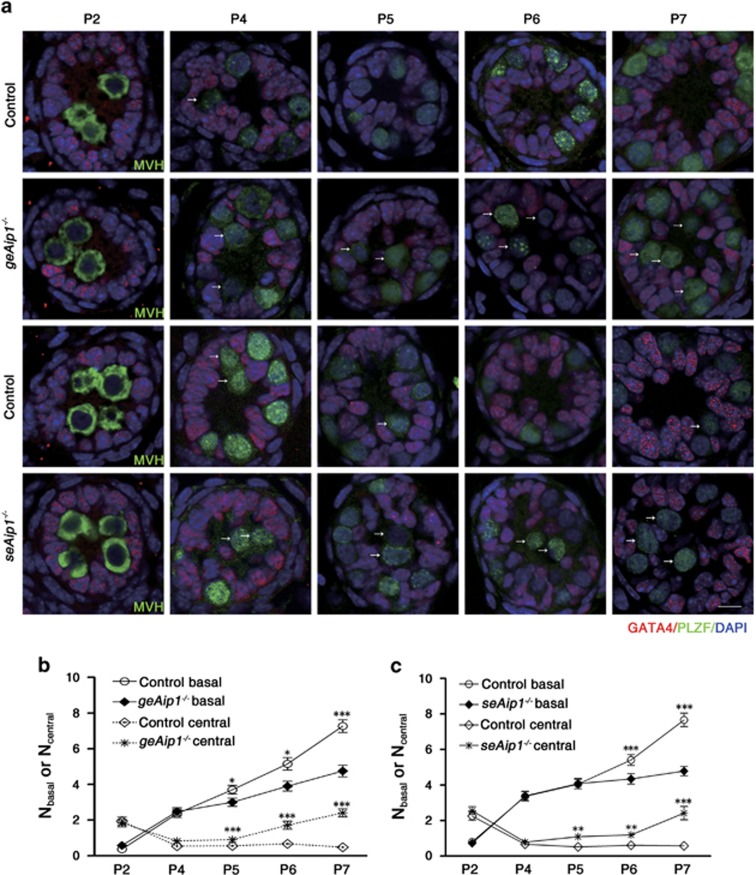
AIP1 is required in both germ cells and Sertoli cells for germ cells migration during postnatal testis development. (**a**) Germ cells from *geAip1*^−/−^ and *seAip1*^−/−^ testes displayed significant defects in their migration as compared with their respective control. Control germ cells (labeled by MVH staining, green) at P2 mostly remained in the central of each tubule, whereas migratory germ cells (labeled by PLZF, green) were observed at P4–P5. By P6 or P7, most of the germ cells had reached and contacted the basement membrane of the testicular cord. In comparison, germ cells in the *geAip1*^−/−^ and *seAip1*^−/−^ testes showed similar localization at P2 and P4 as that of the controls, but displayed significant migration delay starting from P5 or P6. GATA4 staining labels the Sertoli cells (red). White arrows point to examples of germ cells that had not reached the basement membrane. Scale bar: 10 *μ*m. (**b** and **c**) Number of germ cells that were located at either the basal or central region of the testicular cord was quantified in *geAip1*^−/−^ (**b**) and *seAip1*^−/−^ (**c**) testes from P2 to P7 with respect to their controls. Each image was taken from a 5-*μ*m-thick tissue section. A total of 51 tissue cross-sections were analyzed for each genotype at a certain time point (from three mice, except for P2 *geAip1*^−/−^). Data are presented as means±S.E.M.; *P<0.05, **P<0.01, ***P<0.001

We next focused our analysis on the outward migration of germ cells between P2 and P7 for both *geAip1*^−/−^ and *seAip1*^−/−^ testes, as defect in this process is the earliest phenotype we detected and the subsequent severe spermatogenesis phenotypes could be the secondary consequence of earlier defects. To analyze the positioning of germ cells and Sertoli cells within the testicular cord, we immunostained sectioned testis tissues for PLZF (undifferentiated spermatogonia marker^29, 30^), mouse vasa homolog (MVH, general germ cell marker^28^) and GATA4 (Sertoli cell and Leydig cell marker^31^). In P2 control testis, most of round germ cells (gonocytes) labeled with MVH were shown to localize within the center of testicular cord, whereas Sertoli cells were positioned close to the basement membrane. At P4, a major portion of PLZF-labeled germ cells could be seen to relocate to the basement membrane. By P7, most of the germ cells had finished the outward migration, resulting in the large round spermatogonia intermingling with the smaller and irregularly shaped Sertoli cells adjacent to the basement membrane (Figure 3a). To quantify this process of germ cell migration, we counted both the number of germ cells that have reached the basal region of testicular cord (*N*_basal_) and the number of germ cells that have not reached the basement membrane and thus remain in the central region (*N*_central_) for each cross-sectional view of one testicular cord. In the control testes, we found that the total number of germ cells (*N*_central_+*N*_basal_) per section increased from 2.21±0.19 (*n*=51) at P2 to 7.67±0.38 at P7 (*n*=52) (Figure 3b). Such a 3.5-fold increase of germ cell number indicates that postnatal germ cells proliferate during their migration, which is consistent with previous findings.^8^ In the mutant testes, the total number of germ cells increased in a similar fashion as the controls, from 2.28±0.21 at P2 to 7.15±0.41 at P7 (for *geAip1*^−/−^) and from 3.29±0.27 at P2 to 7.24±0.44 at P7 (for *seAip1*^−/−^), respectively (Figure 3b and c), suggesting that *Aip1* deletion in either germ cells or Sertoli cells resulted in no major proliferation defects. Indeed, double staining with Ki67 (proliferative marker) and PLZF for the control and the mutant testes confirmed that no significant proliferation defect occurred in the germ cells in either *geAip1*^−/−^ or *seAip1*^−/−^ testes (Supplementary Figures 2 and 3). However, the *N*_basal_ value, which reflects the extent of germ cell migration, was decreased after P4 or P5 for *geAip1*^−/−^ or *seAip1*^−/−^ testes, respectively. From P2 to P4, there were no significant differences in the *N*_basal_ or *N*_central_ values between the mutant (*geAip1*^−/−^ or *seAip1*^−/−^) and control testes, indicating that germ cells in both mutants migrate normally from P2 to P4. But beginning at P5, *N*_basal_ of *geAip1*^−/−^ testes was significantly decreased as compared with the control, and the decrease became most severe at P7. On the other hand, *N*_central_ value began to display significant increase at P5 for *geAip1*^−/−^ testes (Figure 3b). Similarly, reduction in *N*_basal_ value and increase in *N*_central_ value also took place for *seAip1*^−/−^ testes beginning at P6 and P5, respectively (Figure 3c). Taken together, these results demonstrate that *Aip1* is autonomously required in the germ cells for their migration or relocation to the basement membrane after P4. Interestingly, *Aip1* is also non-autonomously required in the supporting Sertoli cells for germ cell migration. Furthermore, germ cells' migration defects in both *geAip1*^−/−^ and *seAip1*^−/−^ testes were not due to the effects of *Aip1* ablation on cell proliferation of either germ cells or Sertoli cells.

**Figure 4:**
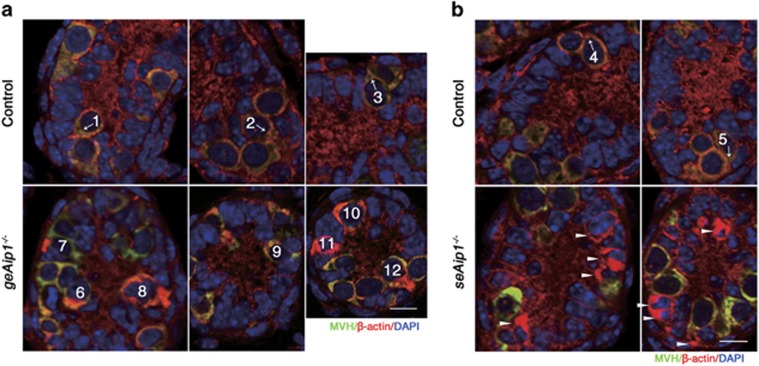
*Aip1* deletion caused ectopic actin accumulations in the cell cortical regions and within lamellipodial protrusions during postnatal testis development. (**a** and **b**) Immunostaining of MVH (green) and *β*-actin (red) of testis tissue sections from P5 control, *geAip1*^−/−^ and *seAip1*^−/−^ mice. The cells numbered 1–5 in the controls are wild-type germ cells migrating toward the basement membrane. Each arrow points to the migration direction and the polarized protrusion of the numbered germ cell. The cells numbered 6–12 are *Aip1*-deleted germ cells containing brightly stained actin patches in cell cortical regions. Arrowheads point to *Aip1*-ablated Sertoli cells with ectopic actin patches. Cell nuclei were stained with DAPI (blue). Scale bars: 10 *μ*m

We next determined what aspects of germ cell migration were prominently affected by *Aip1* deletion in either germ cells or Sertoli cells. Consistent with AIP1's roles in promoting actin dynamics in various model organisms, we found that *Aip1* deletion caused actin disassembly defects and ectopic F-actin patches in Sertoli cells and germ cells within testes of *seAip1*^−/−^ and *geAip1*^−/−^ mice, respectively (Figure 1c and d). The ectopic F-actin phenotype in both *seAip1*^−/−^ and *geAip1*^−/−^ testes began at P4 and became more severe in older mice (Supplementary Figure 4), which is consistent with the increasingly severe migration defects in the mutant testes. Immunostaining with antibody against *β*-actin also verified that the abnormal actin patches were enriched in the mutant testes (Figure 4a and b). In the P5 control testis, *β*-actin staining revealed that a significant portion of germ cells (15 out of 59 in P5 control) that have yet to reach the basement membrane extended polarized and actin-rich lamellipodial protrusions toward the basement membrane (between Sertoli cells or between Sertoli cells and germ cells) during their migration (Figure 4a and B). In P5 *geAip1*^−/−^ testes, these polarized lamellipodial protrusions with membrane-enriched actin staining were much less likely to be observed (15 out of 183). Instead, random (not polarized toward the basal region) protrusions and cell cortical regions filled with intensely stained actin patches were more frequently (62 out of 183) observed in germ cells that have not reached the basement membrane (Figure 4a). At last, in Sertoli cells of *seAip1*^−/−^ testes, cell cortical regions were often observed to be filled with large ectopic patches of intense actin staining beginning at P4 (Figure 4b, Supplementary Figure 4). Together, these data indicate that actin disassembly mediated by AIP1 is required in the cell peripheral and lamellipodial regions of germ cells and Sertoli cells in the wild-type testis to promote outward migration of germ cells.

### *Aip1* deletion caused specific E-cadherin distribution defects in germ cells and Sertoli cells

**Figure 5:**
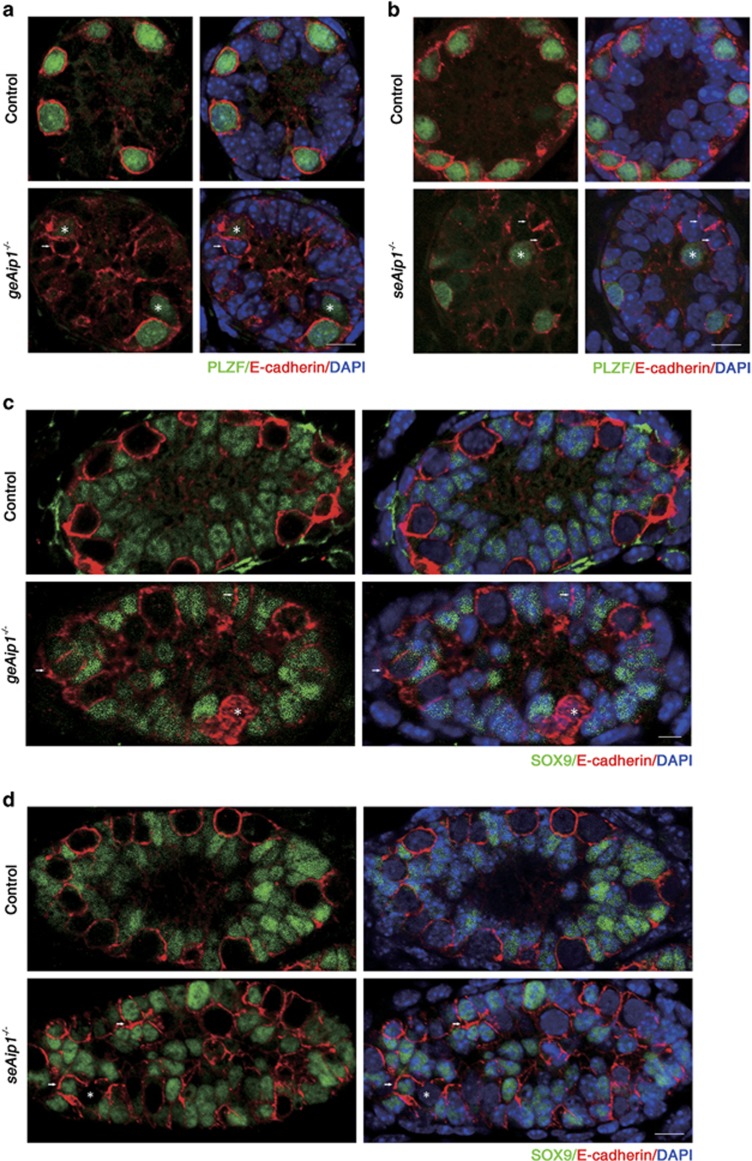
*Aip1* deletion in either germ cells or Sertoli cells caused alterations in E-cadherin distribution pattern in both germ cells and Sertoli cells. (**a** and **b**) Co-immunostaining of E-cadherin (red) and PLZF (green) of testis sections from the P7 control, *geAip1*^−/−^ (**a**) and *seAip1*^−/−^ (**b**) mice. (**c** and **d**) Co-immunostaining of E-cadherin (red) and SOX9 (Sertoli cell marker, green) of testis sections from the P7 control, *geAip1*^−/−^ (**c**) and *seAip1*^−/−^ (**d**) mice. White arrows point to regions of Sertoli cells where E-cadherin level is upregulated, and the asterisk (*) signs mark germ cells whose E-cadherin distribution pattern is affected. Cell nuclei were stained with DAPI (blue). Scale bars: 10 *μ*m

**Figure 6:**
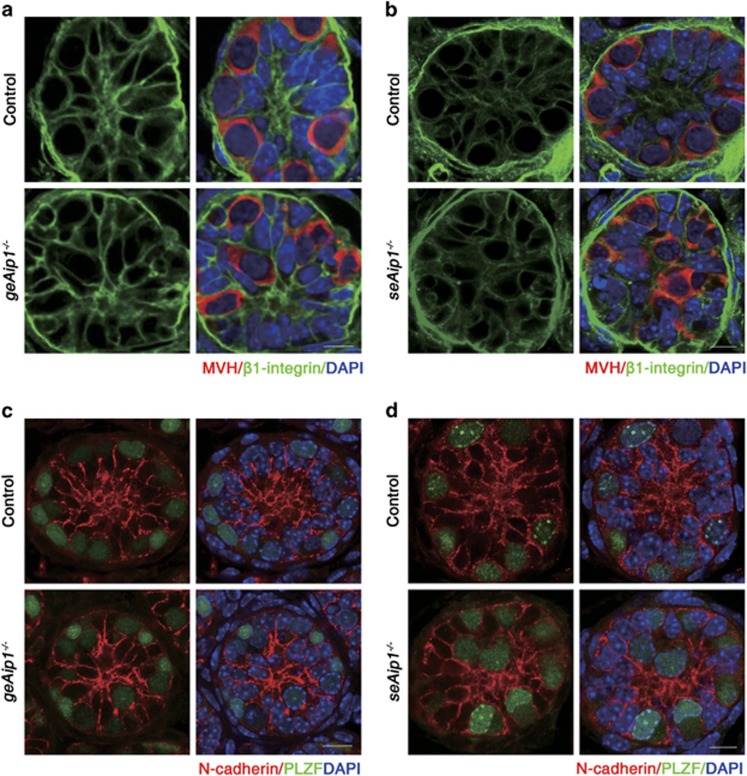
The distribution patterns of N-cadherin and *β*1-integrin were not significantly affected. (**a** and **b**) Co-immunostaining of *β*1-integrin (green) and MVH (red) of testis sections from the P7 control, *geAip1*^−/−^ (**a**) and *seAip1*^−/−^ (**b**) mice. (**c** and **d**) Co-immunostaining of N-cadherin (red) and PLZF (green) of testis sections from the P7 control, *geAip1*^−/−^ (**c**) and *seAip1*^−/−^ (**d**) mice. Scale bars: 10 *μ*m

As cell–cell and cell–substrate adhesions are critical for cell migration, we then examined the distribution patterns of cell adhesion markers in both control and mutant cells. We found that E-cadherin was strongly expressed in PLZF-labeled spermatogonia with a continuous and uniform E-cadherin staining on the cell membrane. In *geAip1*^−/−^ testes, a significant portion (21%, *n*=15 sections from three mice) of P7 germ cells displayed uneven and discontinuous membrane staining on the cell surface. Moreover, ectopic patches of strong E-cadherin staining were found near the cell surface (Figure 5a and c). Intriguingly, such defects of E-cadherin distribution in the germ cells were similarly found in *seAip1*^−/−^ testes (Figure 5b), indicating that AIP1 loss-of-function in Sertoli cells had a non-autonomous effect on germ cells. In addition, we found ectopic E-cadherin staining on the membrane of a significant portion (13.3%, *n*=23) of P7 mutant Sertoli cells (indicated by SOX9, a Sertoli cell marker^32^), whereas control Sertoli cells expressed only very little detectable E-cadherin staining (only 2%, *n*=18) (Figure 5d). Culturing of control and *seAip1*^−/−^ Sertoli cells also confirmed the ectopic increase of E-cadherin level in the mutant Sertoli cells (Supplementary Figure 5). Surprisingly, significant upregulation of E-cadherin was also detected in Sertoli cells in the P7 *geAip1*^−/−^ testes (Figure 5c and Supplementary Figure 6), indicating that *Aip1*-deleted germs cells had a non-autonomous effect on the wild-type Sertoli cells. Next, we found that N-cadherin and *β*1-integrin, two prominent adhesive markers that are uniformly localized on the cell surface of both germ cells and Sertoli cells, distributed normally for both types of cells in *geAip1*^−/−^ and *seAip1*^−/−^ testes (Figure 6a–d). Taken together, these data suggested that the cytoskeletal changes due to AIP1 deficiency caused specific defects in E-cadherin localization for both germ cells and Sertoli cells and that E-cadherin or cytoskeletal defects in one cell type non-autonomously affected E-cadherin distribution of the other cell type.

### *Aip1* deletion in Sertoli cells affected SSC self-renewal and spermatogonial differentiation

**Figure 7:**
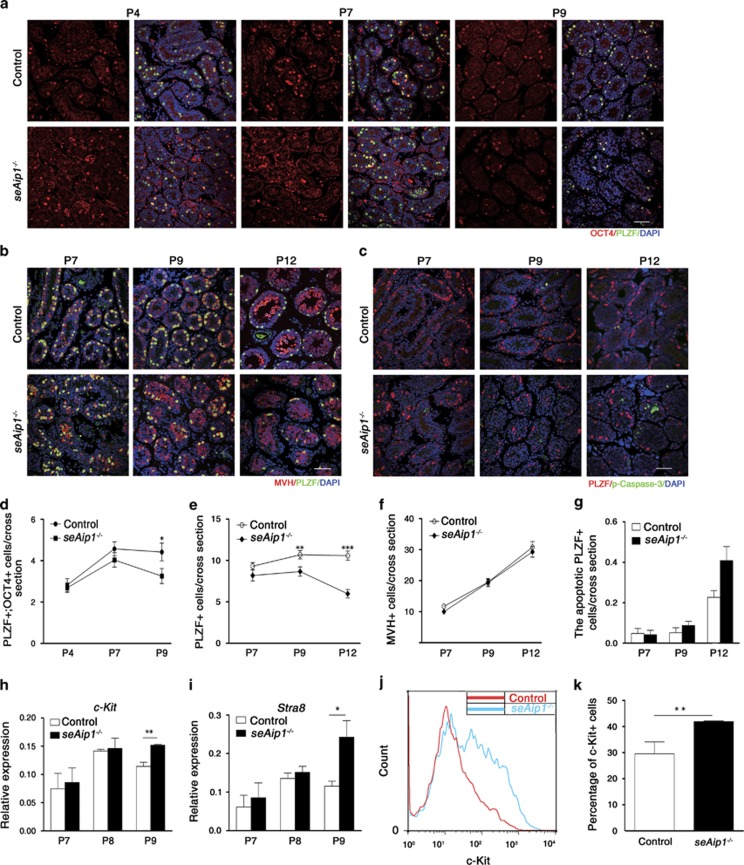
*Aip1* deletion in Sertoli cells resulted in increase of spermatogonial differentiation and decrease of SSC self-renewal. (**a**) Co-immunostaining of OCT4 and PLZF in P4, P7 and P9 *seAip1*^−/−^ and control testes. The result was quantified in (**d**). (**b**) Co-immunostaining of MVH and PLZF in P7, P9 and P12 *seAip1*^−/−^ and control testes, with results quantified in (**e**) and (**f**). (**c**) Co-immunostaining of activated caspase-3 and PLZF in P7, P9 and P12 *seAip1*^−/−^ and control testes, with results quantified in (**g**). (**d**–**g**) Quantification of SSC (**d**; PLZF and OCT double positive), undifferentiated spermatogonia (**e**; labeled by PLZF), germ cells (**f**; labeled by MVH) and apoptotic spermatogonia (**g**; PLZF and activated caspase-3 double positive) per cross-section of each testicular cord for both *seAip1*^−/−^ and control mice at indicated postnatal dates. A total of 51 tissue cross-sections were analyzed for each genotype at a certain time point (from three mice, except for P12 in **c** and **g**). (**h** and **i**) Relative expression levels of *c-Kit* (**h**) and *Stra8* (**i**) from RT-PCR analysis. (**j** and **k**) Increased differentiation as demonstrated by flow cytometry analysis of the c-Kit+ cells. (**j**) Quantification of percentage of c-Kit+ cell from flow cytometry data. (**k**) Scale bars: 50 *μ*m. Data are presented as means±S.E.M.; *P<0.05, **P<0.01, ***P<0.001

The stem cell niche is essential for stem cell functions. To determine the effects of Sertoli cells on self-renewal of SSCs and differentiation of spermatogonia, we examined a variety of stem cell and cell differentiation markers in *seAip1*^−/−^ testes at P4, P7, P9 and P12. We first identified SSCs among undifferentiated spermatogonia in both control and *seAip1*^−/−^ testes by double labeling of PLZF and OCT4 (a marker of SSC in testis^33, 34^). Consistent with previous reports, OCT4 was only expressed in a subset of PLZF-positive cells,^35^ and all OCT4-positive cells are also PLZF-positive. Thus, we considered the OCT4 and PLZF double-positive cells as the SSCs. The number of SSCs in *seAip1*^−/−^ testes was not significantly different from control testes at P4 and P7. However, the number of OCT4 and PLZF double-positive cells as well as the PLZF-positive cells reduced significantly in P9 *seAip1*^−/−^ testes when compared with the control (Figure 7a, b, d and e,Supplementary Figure 7). However, the number of all germ cells that were labeled by MVH (general germ cell marker^36^) remained unchanged in *seAip1*^−/−^ testes at P7, P9 and P12 (Figure 7b and f), suggesting that the reduction of SSCs was specific and was not a secondary consequence of reduction in the general germ cell population. At last, the marked reduction of SSC number at P9 cannot be explained by apoptosis, as there was no significant increase of activated Caspase 3 and PLZF double-positive cells in the P9 or P12 *seAip1*^−/−^ testes (Figure 7c and g). Together, these data indicated that SSCs' self-renewing ability was non-autonomously affected by mutant Sertoli cells.

We next characterized the differentiation profile by real time (RT)-PCR analysis of the expression of *c-kit*,^37, 38, 39^ the marker for differentiating spermatogonia, and *Stra8*,^40^ the marker for pre-meiotic germ cells in the whole testes. We found that the expression of both *c-kit* and *Stra8* increased significantly in *seAip1*^−/−^ testes at P9 (Figure 7h and i). Furthermore, flow cytometry data showed that the percentages of c-Kit+ and PLZF+ cells among the whole testes were significantly increased and decreased, respectively (Figure 7j and k,Supplementary Figure 7), confirming an increase of spermatogonial differentiation and decrease of spermatogonial self-renewal ability in the mutant testes. It was commonly understood that growth factors and chemokines secreted by Sertoli cells and other types of cells in the niche directly regulate the self-renewal and differentiation of SSCs. We next examined the expression levels of a variety of growth factors and chemokines that had been previously shown to affect SSC function. RT-PCR analysis showed that expression levels of *Gdnf*,^41^ *Cxcl12*,^42, 43, 44^ *Fgf2*,^45, 46^ *Egf*^47^ and *Scf*^48, 49^ displayed no significant difference between *seAip1*^−/−^ and control testes (Supplementary Figure 8A). Furthermore, ELISA assay detected no significant alteration in the protein levels of secreted GDNF and FGF2 by isolated Sertoli cells from the P7 *seAip1*^−/−^ testes (Supplementary Figure 8B). At last, transcript levels of *Fshr, Lhcgr, Esr1* and *Ar*, which encode receptors for hormones FSH, LH, estrogen and androgen, respectively,^50, 51^ were not significantly changed in *seAip1*^−/−^ testes except for only mild reduction in levels of *Fshr* at P8 and P9, *Ar* at P9 (Supplementary Figure 8C). These data suggested that the loss-of-function of AIP1 and the resulting SSC niche architecture change did not significantly alter the expression or secretion of growth factors or hormonal receptors in Sertoli cells but rather caused changes in other aspects of the niche microenvironment, which probably includes alterations in the adhesive or architectural properties of the niche. Together, these results indicated that *Aip1* deletion in Sertoli cells resulted in defects in postnatal SSC niche establishment.

### Transplantation restored SSCs from *seAip1*^−/−^ testes to their normal stem cell function

**Figure 8:**
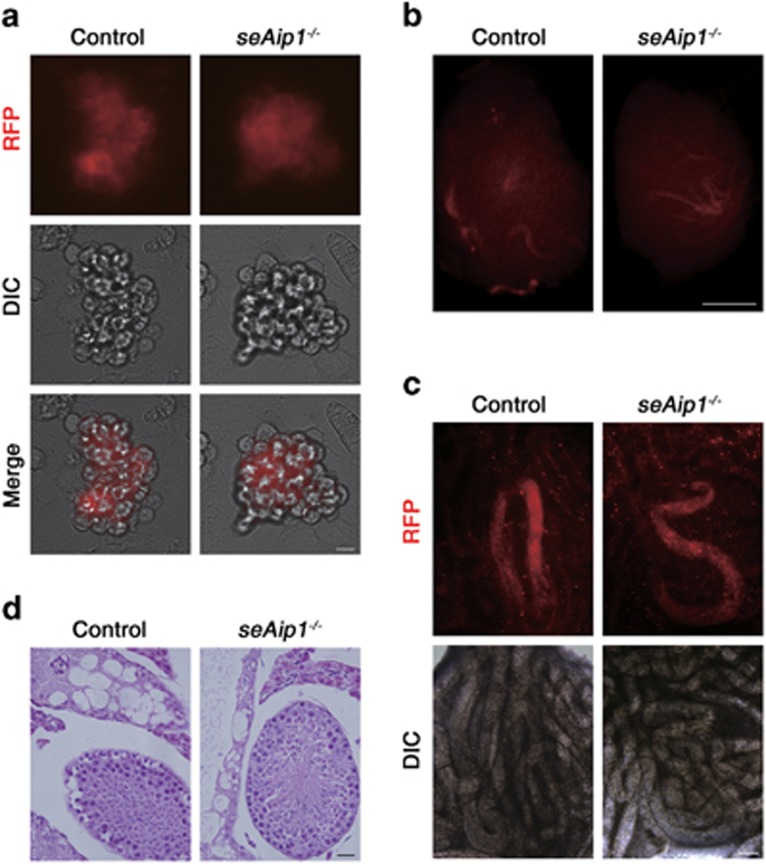
SSCs transplanted from defective niche maintained stem cell function and spermatogenesis potential both *in vivo* and *in vitro*. To trace the donor germ cells, especially in transplantation assay, *seAip1*^−/−^ mice were crossed to *Rosa26-mT/mG* mice, a dual color reporter line, which possesses *lox*P sites on either side of a membrane-targeted *tdTomato (mT)* cassette. After breeding with cre transgenic mice, the resulting offspring would have the *mT* cassette deleted and the downstream membrane-targeted *EGFP (mG)* cassette expressed. (**a**) Representative THY1+ germ cell clumps from testes of *seAip1*^−/−^*:Rosa26-mT/mG* and *Aip1*^*fl/fl*^*:Rosa26-mT/mG* mice that had been passaged for 16 times (2.5 months). (**b**, **c**) Representative images of recipient testes containing mT-labeled colonies (**b**) and mT-labeled seminiferous tubules from recipient testes (**c**). (**d**) Normal spermatogenesis shown by HE staining of the recipient testis that was transplanted with cells from *seAip1*^−/−^*:Rosa26-mT/mG* and *Aip1*^*fl/fl*^*:Rosa26-mT/mG*. Scale bars: 10 *μ*m in (**a**), 1 mm in (**b**), 0.2 mm in (**c**) and 20 *μ*m in (**d**)

To determine whether the disrupted niche in *seAip1*^−/−^ testes could result in irreversible effects on the SSCs, we performed germ cell transplantation, which was considered as effective means to examine the SSC function.^13, 52, 53^ THY1+ germ cells were isolated from P7–P8 testes of *seAip1*^−/−^*:Rosa26-mT/mG* and *Aip1*^*fl/fl*^*:Rosa26-mT/mG* donor mice by magnetic-activated cell sorting (MACS) to enrich SSCs^54, 55^ (Supplementary Figures 9A and B). The busulfan-treated C57BL/6 J × 129sv F1 mice were used as germ cell transplant recipients.^56^ Two months after transplantation with sorted THY1+ cells, the germ cell colonies with red fluorescence (from mT^57^) derived from the donor testes were examined and counted (Figure 8b and c). Surprisingly, germ cells from mutant mice produced 1.5–3.2 folds more colonies in the recipient testes than the control (Table 1). HE staining of the recipient testes showed that there were germ cells of different stages (Figure 8d), indicating that normal spermatogenesis of donor germ cells took place. Furthermore, *in vitro* proliferation assay showed that THY1+ germ cells from mutant testes retained the SSC potential to form colonies and had comparable growth rates as control germ cells (Figure 8a,Supplementary Figure 9). Taken together, these data demonstrated that defects in the niche microenvironment caused by *Aip1* deletion in Sertoli cells did not produce irreversible damage to the SSC function.

## Discussion

SSC's migration, in the context of homing to the germline niche, has recently been extensively studied. This migration event requires RAC1 and *β*1-integrin, but not E-cadherin in the spermatogonia, and it also requires *β*1-integrin in the Sertoli cells.^13, 53^ However, during homing in the adult testis, SSCs need to cross the blood–testis barrier (BTB), which is composed of tight junctions between Sertoli cells.^53^ The migration of gonocytes occurs during the postnatal period and before the inter-Sertoli cells tight junction-BTB establishment (at P12-P14^53^). At present, the underlying mechanism for postnatal germ cell migration is mostly unknown and it is supposed to be different from that of SSC homing. Here, we found that postnatal germ cell migration required AIP1 in both germ cells and Sertoli cells. Ectopic actin accumulations were apparent for both cell types beginning at P4. The onset of the actin defects correlated well with the onset of the migration defects. Our data indicated that impairment of actin disassembly could directly affect postnatal germ cell migration in two major ways.

First, ectopic actin patches were often found in the lamellipodia-like protrusions of migrating *Aip1*^−/−^ germ cells, indicating the functional importance of the actin-based protrusions. A previous study reported that neonatal rat gonocytes with pseudopod extension were identified in the rat testis cell suspensions *in vitro* and these cells could colonize niche of recipient testis much more efficiently than gonocytes without pseudopod.^58^ But similar pseudopod extensions in gonocytes had not been detected in mouse.^9^ Our study provided important *in vivo* evidence that actin-rich protrusions existed in the postnatal germ cells and that these protrusions probably required robust actin turnover to drive germ cells' migration toward the basement membrane.

Second, the lack of actin dynamics could disrupt E-cadherin-mediated cell adhesion, leading to block of germ cells migration. Our data showed that the distribution pattern of E-cadherin but not of N-cadherin and *β*1-integrin was significantly affected in *Aip1*^−/−^ germ cells and Sertoli cells. It is known that the membrane distribution and clustering of E-cadherin molecules are regulated by the dynamics of the underlying actin network. We have previously shown that the lack of AIP1 function in *Drosophila* caused disruption of E-cadherin remodeling and distribution in the adherens junction, often resulting in discontinuous membrane staining between two mutant cells. A recent study done in *Drosophila* testis revealed that disturbing actin polymerization by removing the function of profilin, an actin polymerization-promoting factor, also caused disruption of E-cadherin staining in the interface between GSCs and the supporting hub cells.^17^ In agreement with the above reports, our data indicated that *Aip1* deletion resulted in the disruption of E-cadherin distribution in the interface between germ cells and Sertoli cells, which could lead to their migration defects. Interestingly, *Aip1* deletion in Sertoli cells also led to ectopic patches of E-cadherin staining near the cell membrane of Sertoli cells as well as discontinuous E-cadherin staining (but without ectopic patches) in the cell membrane of the spermatogonia. The autonomous effect of strong increase of membrane-associated or ectopically located E-cadherin could be explained if robust actin dynamics is required to promote fast turnover of membrane-associated E-cadherin in the wild-type Sertoli cells (probably via endocytosis). Indeed, actin dynamics has been shown to be critical for endocytosis.^59^ Furthermore, the non-autonomous effect on spermatogonia's E-cadherin distribution could be due to the altered pattern of E-cadherin localization in the adjacent Sertoli cells and the homophilic nature of the trans-membrane E-cadherin.

Our data from *Aip1* deletion in the Sertoli cells also provided important insights into how the SSC niche in the neonatal testis is established and how the niche influences SSC self-renewal and differentiation. The niche is commonly thought of consisting of two parts, namely a growth factor milieu and architectural support.^1^ The Sertoli cell-specific ablation of *Aip1* did not significantly alter the expression and secretion levels of growth factors in the Sertoli cells. However, germ cell migration toward the basement membrane was impaired, beginning at P5. This result suggested that disruption of germ cells migration results in defects in the establishment of the initial (neonatal) SSC niche, eventually leading to impairment of SSC function. This is possibly caused by the changes in niche architecture, which were manifested as alterations in the actin distribution in Sertoli cells and E-cadherin distribution in both the supporting Sertoli cells and spermatogonia. Interestingly, the architectural and adhesive alterations in the niche did not result in a permanent impairment of SSC function, as transplanting the SSCs taken from *seAip1*^−/−^ testes can efficiently re-colonize recipient testes. This result suggested that disruption of SSC function as a result of architectural changes in the postnatal niche is reversible and that the *seAip1*^−/−^ mice could serve as a good model to study the underlying mechanisms.

## Materials and Methods

### Mice

Mice were housed in standard cages in an Assessment And Accreditation of Laboratory Animal Care accredited SPF animal facility on a 12 h light/dark cycle. All animal protocols are approved by the Animal Care and Use Committee of the Model Animal Research Center of Nanjing University, the host for the National Resource Center for Mutant Mice in China. All mice used in this study were of a mixed 129/B6 background.

### Histology and immunofluorescence

Testes were fixed with 4% paraformaldehyde, embedded in paraffin and transversely sectioned at a thickness of 5 *μ*m. Tissue sections were stained with HE to examine histology. Immunofluorescence was carried out as previously described.^60^ Sections were counterstained with DAPI (Sigma-Aldrich, Saint-Quentin Fallavier, France), mounted in vectashield aqueous medium (Vector Laboratories, Inc., Burlingame, CA, USA), and analyzed using Olympus FV1000 confocal microscope. Primary antibodies used are: rabbit-anti-SOX9 (The original concentration is 200 *μ*g/ml, 1 : 50), mouse-anti-PLZF (200 *μ*g/ml, 1 : 200), mouse-anti-*β*-actin (100 *μ*g/ml, 1 : 200), mouse-anti-GATA4 (200 *μ*g/ml, 1 : 200), rabbit-anti-PLZF (200 *μ*g/ml, 1 : 200), mouse-anti-OCT4 (200 *μ*g/ml, 1 : 100) (Santa Cruz Biotechnology, Inc., Dallas, TX, USA, sc-20095, sc-28319, sc-81178, sc-25310, sc-22839, sc-5279), rabbit-anti-MVH (0.8 mg/ml, 1 : 300), rabbit-anti-STRA8 (0.8 mg/ml, 1 : 300) (Abcam, Cambridge, UK; Ab13840, Ab49602), Rat-anti-Ki67 (0.74 mg/ml, 1 : 200) (Dakocytomation, Glostrup, Denmark; M7249), rabbit-anti-Cleaved caspase-3 (79 *μ*g/ml, 1 : 200) (Cell Signaling Technology Inc., #9661), mouse-anti-E-cadherin (250 *μ*g/ml, 1 : 200), mouse-anti-N-cadherin (250 *μ*g/ml, 1 : 200) (BD Biosciences, San Jose, CA, USA; 610182, 610920), rat-anti-*β*1-integrin (100 *μ*g/ml, 1 : 100) (EMD Millipore, MAB1997; Billierca, MA, USA). Cy3- Cy5- and FITC-conjugated antibodies (Jackson ImmunoResearch Laboratories, West Grove, PA, USA; 115-165-146 (1 mg/ml, 1 : 200), 111-165-003 (0.75 mg/ml, 1 : 200), 715-175-150 (0.25 mg/ml, 1 : 200), 114-095-146 (0.75 mg/ml, 1 : 200), 111-095-144 (0.75 mg/ml, 1 : 200)) and AlexaFluro 488-conjugated antibodies (Invitrogen, Cergy Pontoise, France, A21208 (2 mg/ml, 1 : 200)) were used as secondary antibodies. F-actin was labeled by AlexaFluor555 phalloidin (Invitrogen; A34055 (200 unit/ml, 1 : 200)).

### Isolation and culture of THY1+ germ cells

Mouse testes were collected and digested by two-step enzymatic digestion procedure using collagenase and trypsin as described.^52, 61^ In brief, after removing tunica albuginea of P7–P8 mouse testes, testicular cords were incubated with type IV collagenase (1 mg/ml; Sigma) in 37 °C for 10 min, following trypsin (0.25%) and DNase (7 mg/ml) incubation for 5 min. MACS was used to isolate THY1+ germ cells (Miltenyi Biotec, Bergisch Gladbach, Germany; anti-CD90.2, 130–049–101) as previously described.^62^ Cells were plated into wells of 12-well plate on mitotically inactivated STO (SIM mouse embryo-derived thioguanine and ouabain resistant) cell feeders (5 × 10^4^ cells/cm^2^) as described.^61, 62^ Cells were cultured with previously described optimized serum-free medium.^61^ All cultures were maintained at 37 °C in a humidified 5% CO_2_ incubator. The medium was changed every 2–3 days.

### Flow cytometry

The experiment was carried out as previously described.^43^ In brief, the single-cell suspension of the P9 testes cells derived from two-step enzymatic digestion were incubated with the Anti-Mouse CD117 (c-Kit) APC-eFluor 780 antibody (0.125 *μ*g/test) (eBioscience, Inc., San Diego, CA, USA). For the PLZF FACS, the P9 testes cell suspensions were first fixed and washed using the transcription factor buffer set (BD Biosciences, 562574) before incubated with PLZF (D-9) (0.2 *μ*g/test) (sc-28319), then the Cy5-conjugated Goat-anti mouse antibody (Jackson ImmunoResearch Laboratories, 715-175-150 (0.5 *μ*g/ml, 1 : 200)) were incubated after washing by wash/perm provided in the buffer set. The stained cells were analyzed by FACS Calibur (BD Biosciences).

### *In vitro* proliferation assay

After initial seeding density at 2 × 10^5^ cell/well in 12-well plates, cultured THY1+ cells with a typical clump-forming morphology after 6 days culture were gently blew off from STO feeder layer and collected into a 15-ml conical tube. After rinse and centrifugation, cells were dissociated with Trypsin-EDTA to achieve a single-cell suspension. Following three washes in HBSS buffer, cells were concentrated and counted with Hemocytometer under microscope.

### Transplantation assay

*Aip1*^*fl/fl*^*:Amh*^*+/cre*^*:Rosa26-mT/mG* and *Aip1*^*fl/fl*^*:Rosa26-mT/mG* mice were used as the mutant and control donors. MACS THY1+ cells were isolated from 12 testes of six mice at the age of P7–P8. 2.5 × 10^4^ THY1+ SSCs were transplanted into one C57BL/6 J × 129sv F1 recipient testis, which were treated with busulfan at 6–8 weeks of age and received donor cells 2 months after busulfan treatment. The colony number was analyzed under the NIKON ECLIPSE E800 fluorescence microscopy.

### RNA extraction and RT-PCR

The *geAip1*^−/−^, *seAip1*^−/−^ and control mouse testis were stripped of the tunica albuginea, placed in liquid nitrogen. Total RNAs were extracted using Trizol (Invitrogen, Carlsbad, CA, USA) according to the manufacturer's protocol. Three microgram of RNA was used for First-strand cDNA synthesize using RevertAid First Strand cDNA Synthesis Kit (Fermentas, Burlington, ON, USA). To quantify mRNA expression using RT-PCR, comparisons were made by normalizing the expression to that of glyceraldehyde-3-phosphate dehydrogenase (*Gapdh*) using Power-SYBR Green PCR Master mix (Takara, Takara Island, Japan) and StepOnePlus Real-Time System (ABI system, Cape Coral, FL, USA). The PCR conditions were 95 °C for 30 s, followed by 40 cycles of 95 °C for 5 s, 60 °C for 31 s. PCR was carried out using the primers listed in the Supplementary Table 1.

### Sertoli cell isolation and ELISA

The Sertoli cells were isolated according to the protocol as previously described.^63, 64^ in brief, digested testes cells were cultured in DMEM medium (Sigma-Aldrich) that was supplemented with sodium bicarbonate (1.2 mg/ml) and 10% fetal calf serum (Life Technologies, Carlsbad, CA, USA). After 24 h incubation in a humidified atmosphere at 37 °C with 5% CO_2_, the cells were treated with a hypotonic solution (0.3 × HBSS, pH 7.4) for 3 min to remove the spermatogenic cells adhering to the Sertoli cells. Then serum-free DMEM was added. After 24 h culturing, the medium was collected and concentrated. Elisa was performed using the concentrated sample and FGF2 Elisa kit (15.6 pg/ml~1000 pg/ml, E90551 Mu, USCN, Wuhan, China) and GDNF Elisa kit (78 pg/ml~5000 pg/ml, E90043 Mu, USCN).

### Testicular cell co-culture

The experiment was performed as previously described with small modifications.^65, 66^ In brief, the testicular cells from P4 *seAip1*^−/−^*:Rosa26-mT/mG* and *Aip1*^*fl/fl*^*:Rosa26-mT/mG* mice were isolated, three male pups for each group. And the testes were decapsulated in cold PBS, then collected and treated by the following sequential enzymatic digestion: 0.1% collagenase–0.05% hyaluronidase–0.1 mg/ml DNase for 20 min, 0.1% collagenase–0.1 mg/ml for another 20 min, 0.05% Trypsin-EDTA for 2 min. After washing the cell suspension by trypsin inhibitor BSA (0.65% and 0.05%, respectively), the cells were plated at a density of 4.8 × 10^3^ cells/mm^2^ in the 24-well plate that had been pre-coated with gelatin and were cultured at 37 °C in a 5% CO^2^ atmosphere. The culture medium was DMEM supplemented with penicillin, streptomycin, 1 mM Na pyruvate, 1 × nonessential amino acids, 3 mM Na lactate, 5 *μ*g/ml transferrin and 50 ng/ml retinol. The medium was changed after 12 h plating and every 2 days thereafter.

### Western blot

The THY1+ germ cells were harvested using the protocol described in the section of ‘Isolation and Culture of THY1+ Germ Cells', and Sertoli cells were harvested using protocol described in ‘Sertoli cell isolation and Elisa'. Sertoli cells that were cultured *in vitro* for 24 h and isolated THY1+ cells were collected and lysed with lysis buffer (1% NP-40, 0.5% sodium deoxycholate, 0.1% SDS, 150 mM sodium chloride, 0.1 mM EDTA, 50 mM Tris-HCl, pH 7.2, supplemented with 1 mM NaF, 1 mM NaVO4, 1 mM PMSF and Protease inhibitor cocktail (P3840, Sigma-Aldrich, St Louis, MO, USA)). Proteins concentration was measured using the BCA Protein Assay kit (23255, Pierce, Chicago, IL, USA). In total, 2–3 *μ*g of protein extracts were analyzed by immunoblotting with the following primary antibodies: rabbit-anti-AIP1 (22 *μ*g/150 *μ*l, 1 : 500) (Protein tech group, Chicago, IL, USA; 13676-1-AP), mouse-anti-GAPDH (100 *μ*g/ml, 1 : 1000) (sc-32233), mouse-anti-PLZF (D-9) (200 *μ*g/ml, 1 : 500) (sc-28319). HRP-conjugated secondary antibodies were used (Pierce, 31430 (0.8 mg/ml, 1 : 2000), 31460 (0.8 mg/ml, 1 : 2000)).

## Figures and Tables

**Table 1:** Transplantation result

	**Control**	***seAip1***^−/−^	**Fold change**
*Experiment 1*			
Colony number/10^5^ THY1+ cells	1.19	3.8	3.2
Colony length (mm)/10^5^ THY1+ cells	1.30	5.24	4
			
*Experiment 2*			
Colony number/10^5^ THY1+ cells	5.96	9.23	1.55
Colony length (mm)/10^5^ THY1+ cells	10.7	13.14	1.23
			
*Experiment 3*			
Colony number/10^5^ THY1+ cells	18.67	27.64	1.48
Colony length (mm)/10^5^ THY1+ cells	31.3	57.25	1.83

The total number of colonies from three to six different recipient testes was analyzed for each group

## Abbreviations

SSCs: spermatogonial stem cells
AIP1: actin-interacting protein 1
F-actin: actin filaments
PGCs: primordial germ cells
GSCs: germline stem cells
ADFs: actin-depolymerizing factors
HE: hematoxylin and eosin
MACS: magnetic-activated cell sorting
BTB: blood–testis barrier
