# Supplementary material for: AIP1-mediated actin disassembly is required for postnatal germ cell migration and spermatogonial stem cell niche establishment
Source: Cell Death Dis. 2015 Jul 16;6(7):e1818–. doi: 10.1038/cddis.2015.182 (PMC4650729; doi:10.1038/cddis.2015.182)
Supplement: Supplementary Information [file cddis2015182x1.doc]

**Supplemental Figures and Table**

**
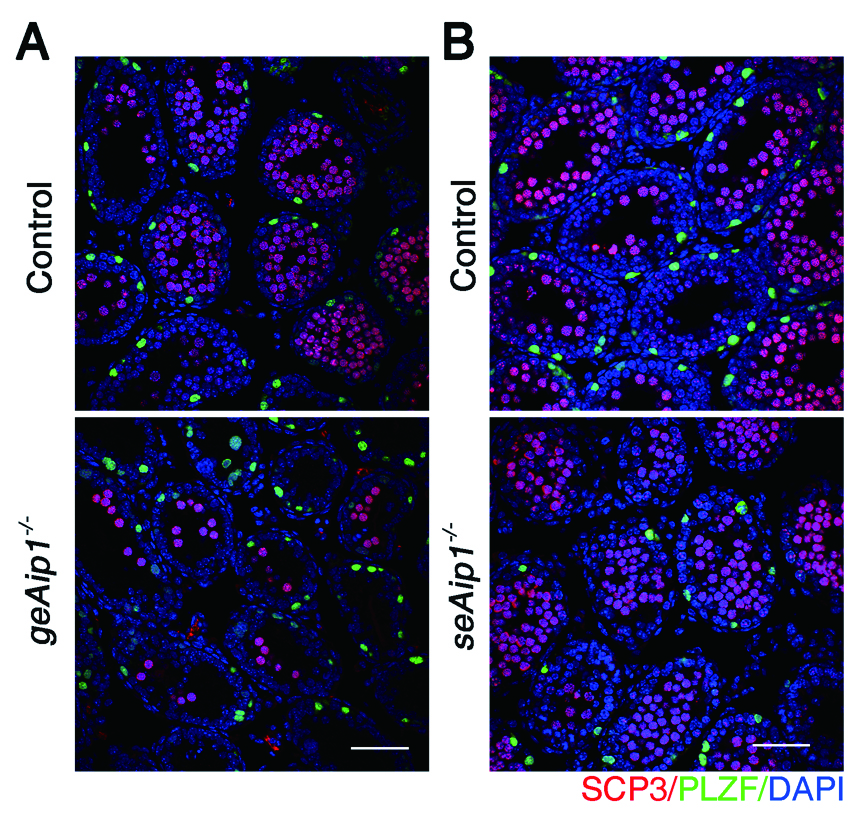
**

**Supplementary Figure 1** The number of primary spermatocytes was markedly reduced in *geAip1^-/-^* testes but not in *seAip1^-/-^* testes at P12. (A, B) Co-immuostaining of meiosis I specific marker SCP3 (red) and PLZF (green) of testis sections from control, *geAip1^-/-^* and *seAip1^-/-^* testes. SCP3 positive spermatocytes were markedly reduced in *geAip1^-/-^* testes as compared to the control (A). The number of SCP3 positive spermatocytes was comparable between control and *seAip1^-/-^* testes (B). Cell nuclei were stained with DAPI (blue). Scale bars: 50 μm.


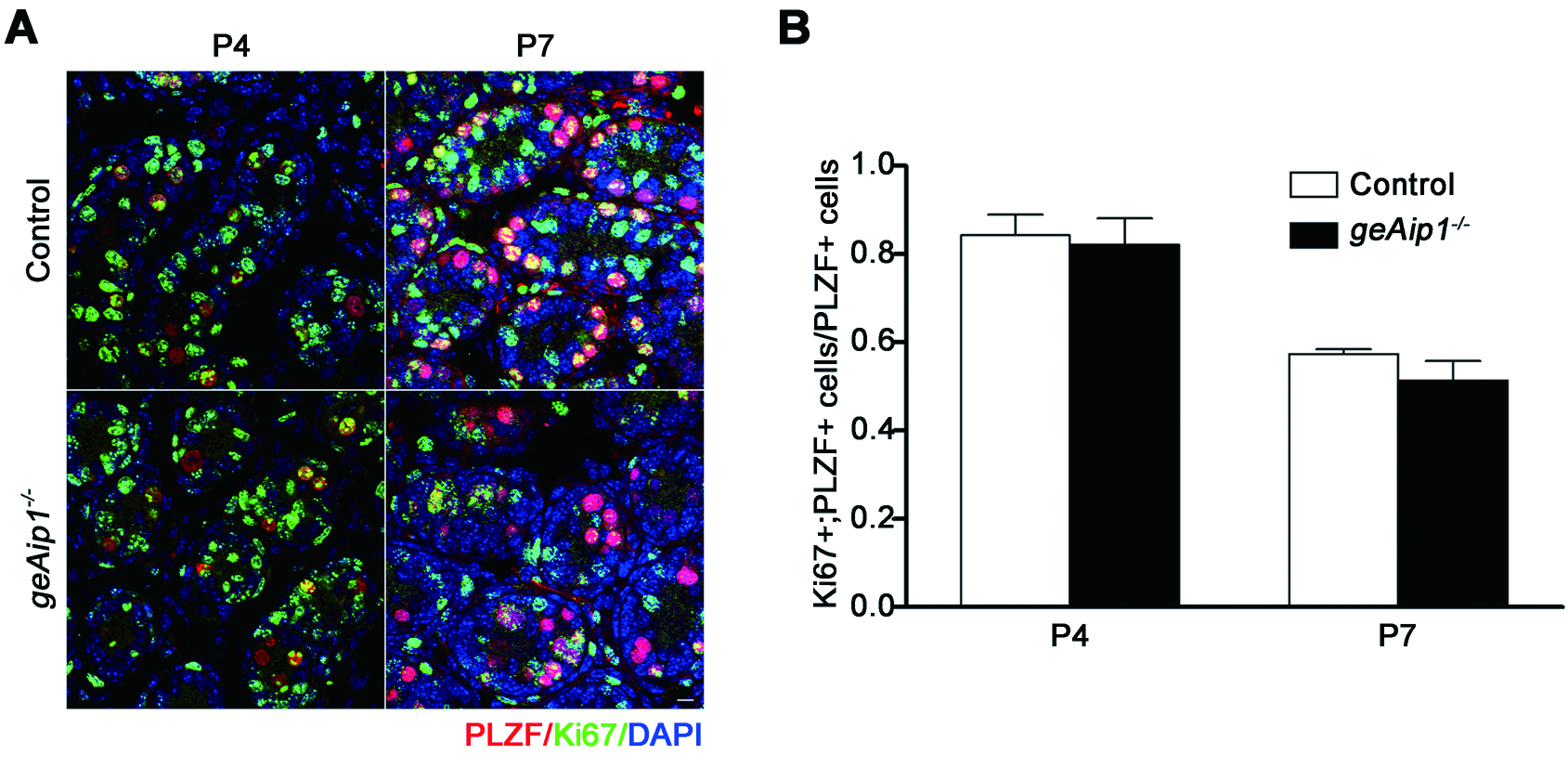


**Supplementary Figure 2**  Proliferation of early germ cells was not affected in *geAip1^-/-^* testis. (A) The proliferation of undifferentiated spermatogonia in the *geAip1^-/-^* mice as shown by co-immunostaining of Ki67 (green) and PLZF (red) was not affected at P4 and P7. (B) The ratio of proliferating spermatogonia among all PLZF-labeled spermatogonia in *geAip1^-/-^* testes was not significantly altered as compared to the control. Cell nuclei were stained with DAPI (blue). Scale bar: 10 μm. Data are presented as means±S.E.M.; *n*=3.


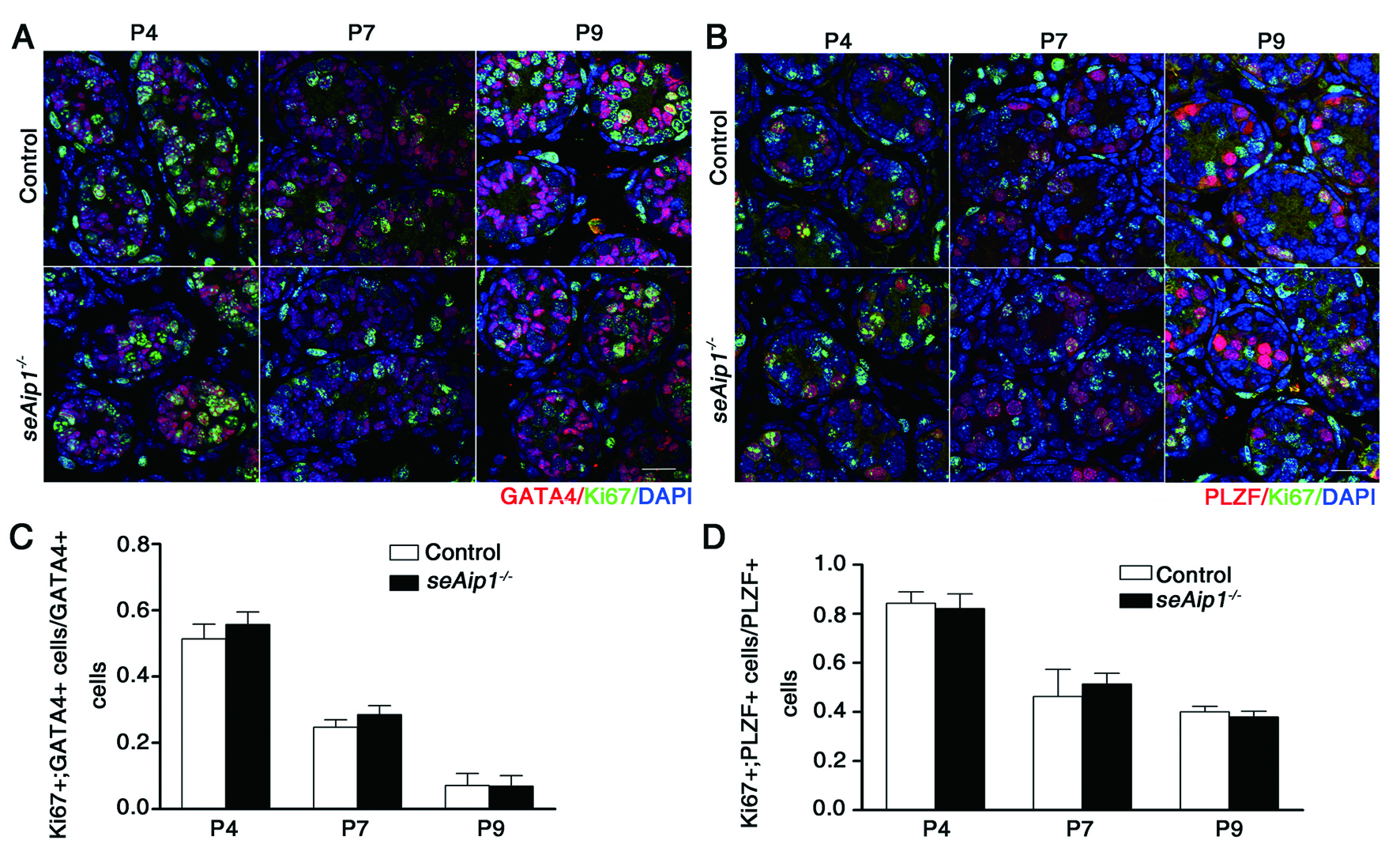


**Supplementary Figure 3** Proliferation of early Sertoli cells and germ cells was not affected in *seAip1^-/-^* testis. (A, C) The proliferation of Sertoli cells in the *seAip1^-/-^* mice as shown by co-immunostaining of GATA4 (red) and Ki67 (green) was not affected at P4, P7 and P9 (A). The ratio of proliferating Sertoli cells among all GATA4-labeled Sertoli cells in *seAip1^-/-^* testes was not significantly altered as compared to the control (C). (B, D) The proliferation of spermatogonia in the *seAip1^-/-^* mice as shown by co-immunostaining of Ki67 (green) and PLZF (red) was not affected at P4, P7 and P9 (B). The ratio of proliferating spermatogonia among all spermatogonia in *seAip1^-/-^* testes was not significantly altered as compared to the control (D). Cell nuclei were stained with DAPI (blue). Scale bars: 20 μm. Data are presented as means±S.E.M.; *n*=3.

**
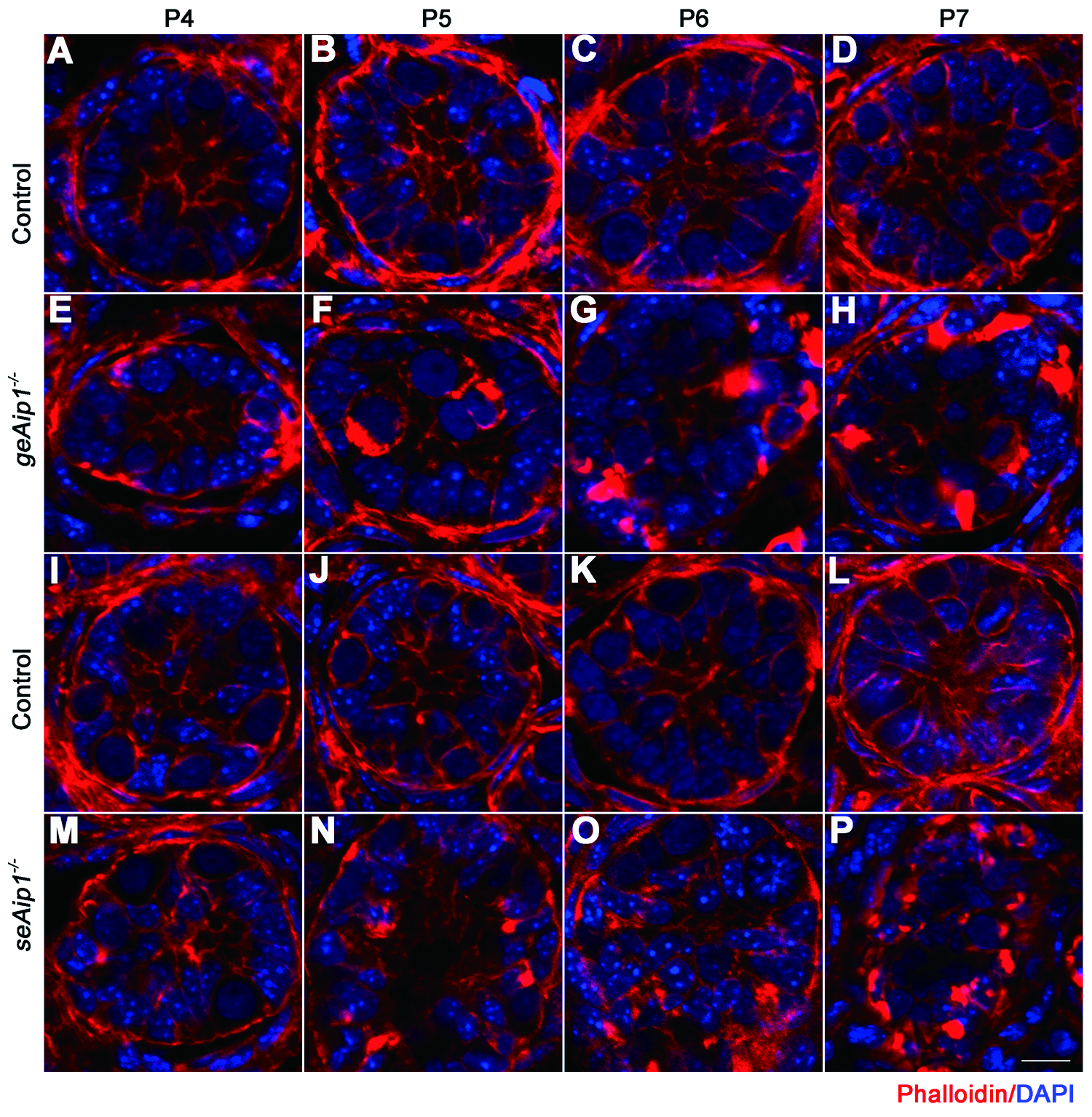
**

**Supplementary Figure 4** Time course analysis of effect of *Aip1* deletion on F-actin levels in Sertoli cells and germ cells. (A-P) Representative images of phalloidin staining (red) of testes from the control (A-D, I-L), *geAip1^-/-^* (E-H), and *seAip1^-/-^* (M-P) mice. Cell nuclei were stained with DAPI (blue). Scale bar: 10 μm.


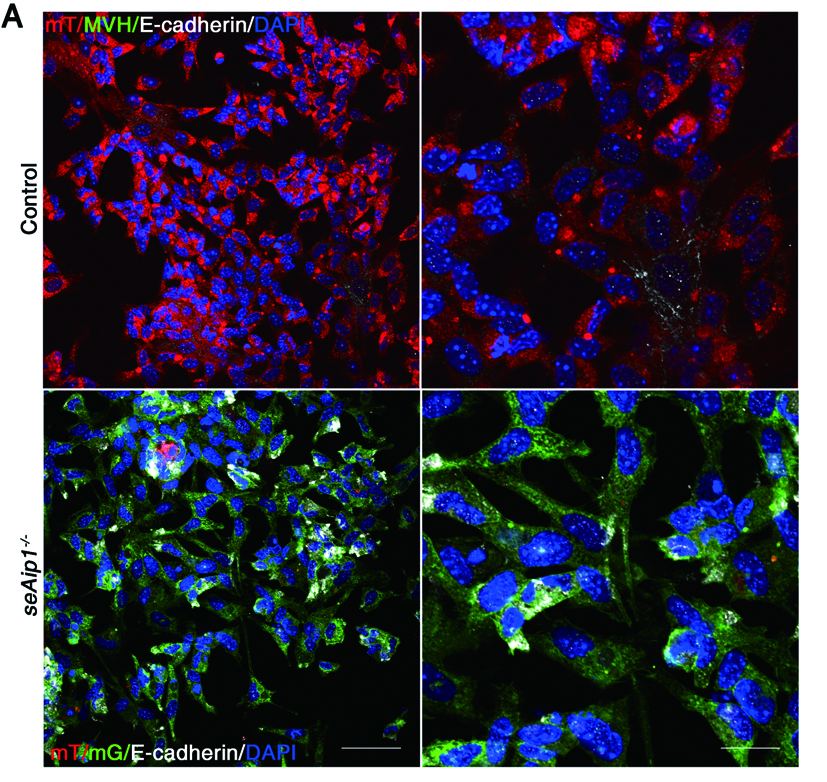


**Supplementary Figure 5** Increased E-cadherin expression in cultured *Aip1*-deleted Sertoli cells. (A) To further confirm the ectopic increase of E-cadherin level in the mutant Sertoli cells, we cultured the Sertoli cells along with germ cells from P4 *Aip1^fl/fl^:Amh^+/cre^:Rosa26-mT/mG* (*seAip1^-/-^*) and *Aip1^fl/fl^:Rosa26-mT/mG* (Control) testes. Most of the germ cells were lost from cell culture after 9 days of culturing, and most of the remaining cells were Sertoli cells and they would be free from the influence from germ cells. We found that after 12 days of culturing, Sertoli cells from control testes displayed little E-cadherin staining, similar to what was observed *in vivo*. In contrast, a major portion of *Aip1*-deleted Sertoli cells in cell culture displayed significant increase of overall E-cadherin level and ectopic E-cadherin patches in the cell cortex and underlying cytoplasmic regions, which were also observed in Sertoli cells within the *seAip1^-/-^* testes. *Rosa26-mT/mG* is a dual color reporter containing *lox*P sites on either side of a membrane targeted *tdTomato (mT)* cassette. Cells from control testes only displayed mT (red) fluorescence, whereas Sertoli cells from *seAip1^-/-^* testes displayed mG (GFP, green) due to the action of Cre, which would make the *mT* cassette deleted and the downstream membrane targeted *GFP* *(mG)* cassette expressed. Immunostaining of E-cadherin demonstrated that E-cadherin expression was markedly increased in *Aip1*-deleted Sertoli cells. Cell nuclei were stained with DAPI (blue). Scale Bars: 50 μm in the left panels and 20 μm in the right panels.


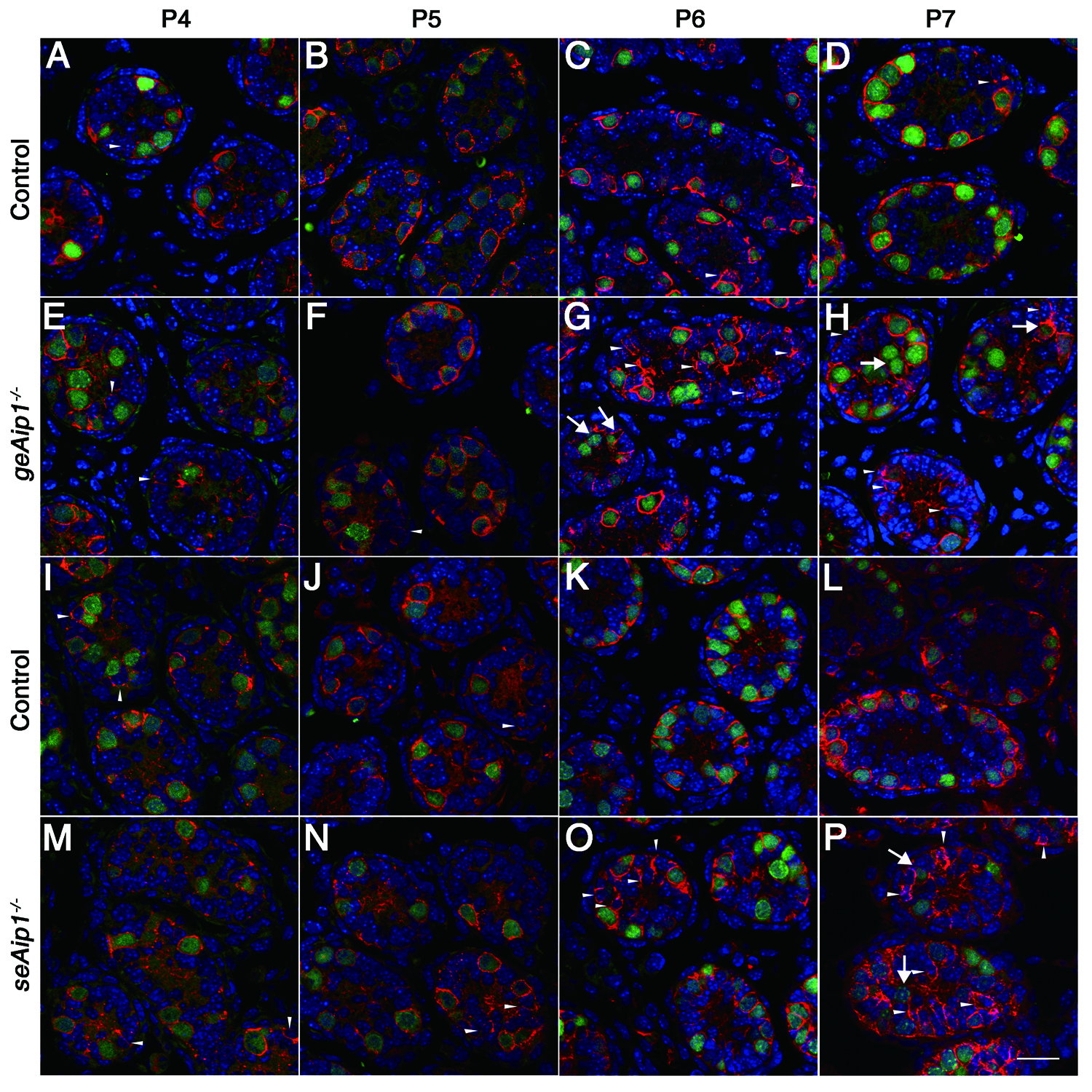


**Supplementary Figure 6** Time course analysis of E-cadherin expression demonstrates increasingly E-cadherin distribution defects in both germ cells and Sertoli cells during postnatal testis development. (A-P) Representative images of E-cadherin (red) and PLZF (green) staining of testes from the control, *geAip1^-/-^* and *seAip1^-/-^* mice from P4 to P7. White arrowheads point to regions of Sertoli cells where E-cadherin level is up-regulated (beginning at P6), the arrows mark germ cells whose E-cadherin distribution pattern is affected. Cell nuclei were stained with DAPI (blue). Scale bar: 20 μm


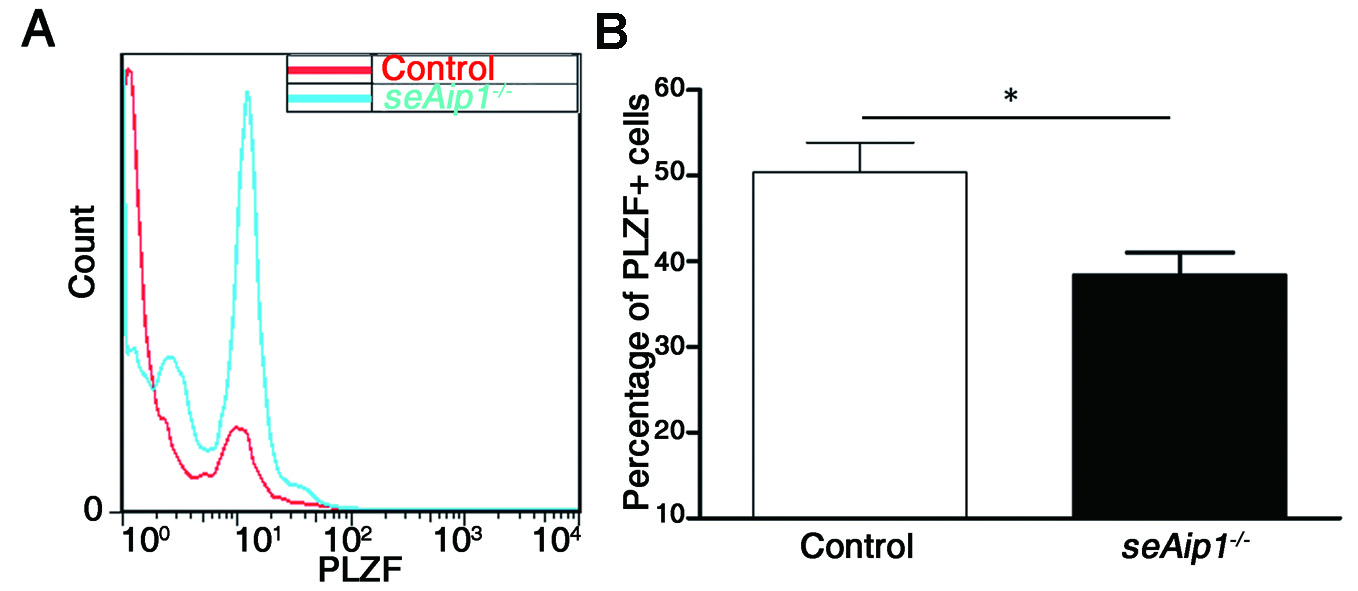


**Supplementary** **Figure 7** *Aip1* deletion in Sertoli cells resulted in decrease of undifferentiated spermatogonia**.** (A) Representative flow cytometry analysis of the PLZF+ cells in *seAip1^-/-^* mice as compared to the control. (B) Quantification of percentage of PLZF+ cells from flow cytometry data. Data are presented as means±S.E.M.; *n*=3; **P*<0.05.


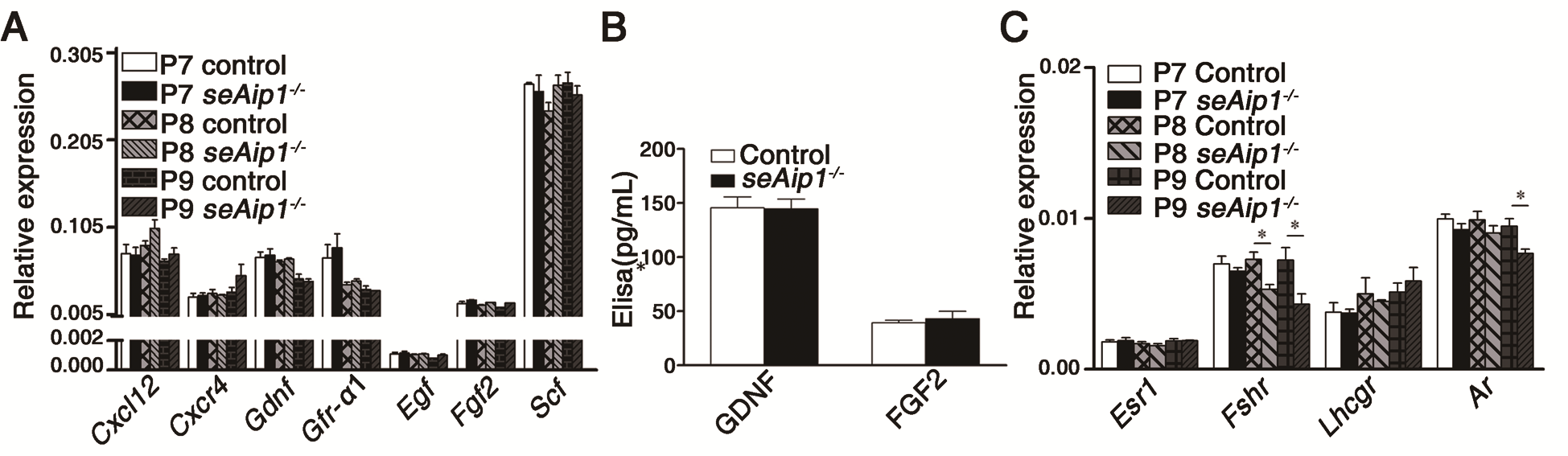


**Supplementary Figure 8** The expression of genes encoding a number of growth factors and receptors was not changed in *seAip1^-/-^* testis. (A) RT-PCR analysis of the expression of genes encoding growth factors and some of their receptors in P7-P9 control and *seAip1^-/-^* testes. Note that *Gfr-α1* and *Cxcr4* encode for the receptors for GDNF and CXCL12 respectively. (B) Elisa analysis of the secretion of GDNF and FGF2 by Sertoli cells from P7 control and *seAip1^-/-^* mice. (C) RT-PCR analysis of the expression of genes encoding the hormonal receptors in P7-P9 control and *seAip1^-/-^* testes. Data are presented as means±S.E.M.; *n*=3; **P*<0.05.


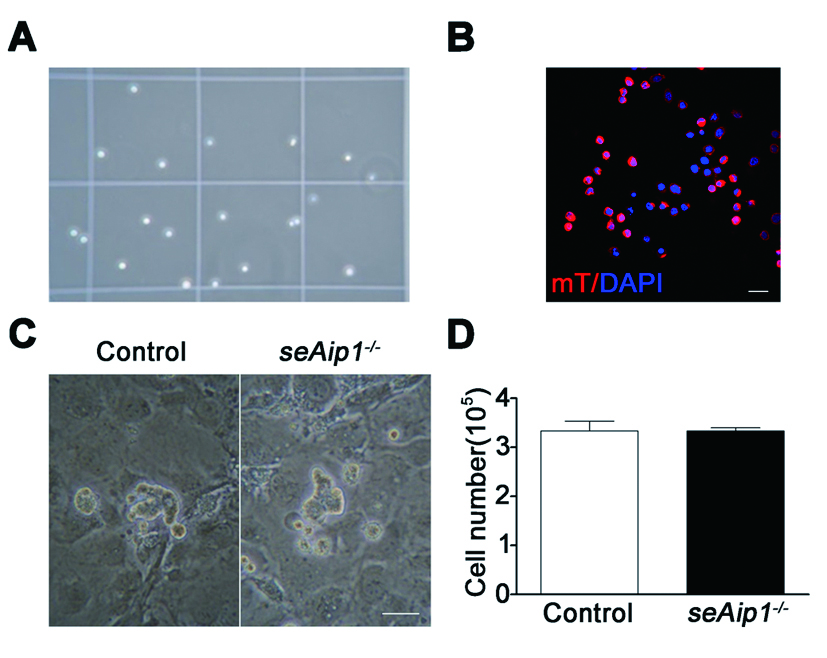


**Supplementary Figure 9** THY1+ germ cells taken from defective niche maintain stem cell potential *in vitro*. (A) Representative image of MACS THY1+ cells. (B) mT-marked THY1+ cells from testes of *seAip1^-/-^:Rosa26-mT/mG* mice showed successful isolation of THY1+ cells in which SSCs were enriched. (C) THY1+ cells isolated from P7 *seAip1^-/-^:Rosa26-mT/mG* testes which had been cultured for 6 days displayed normal clump forming efficiency (indicated by the clump number and size) as comparable to the Control. (D) The proliferation of the SSCs was determined by counting their numbers 6 days after seeding 2×10^5^ cells on STO feeder cells. THY1+ germ cells from *seAip1^-/-^:Rosa26-mT/mG* testes had comparable growth rates as control germ cells. Cell nuclei were stained with DAPI (blue). Scale bars: 20 μm. Data are presented as means±S.E.M.; *n*=3.

**Supplementary Table 1**  RT-PCR primers

| Gene | Forward primer (5'-3') | Reverse primer (5'-3') |
| --- | --- | --- |
| *Gapdh* | CATGGCCTTCCGTGTTCCT | GCGGCACGTCAGATCCA |
| *Stra8* | GTTTCCTGCGTGTTCCACAAG | CACCCGAGGCTCAAGCTTC |
| *c-kit* | TCGGTGCACTTGGGCGAGAG | ACGCAGAGCAGATCCCAGGC |
| *Gdnf* | ATTTTATTCAAGCCACCATTA | GATACATCCACACCGTTTAGC |
| *Gfrα1* | TGCGTATCTACTGGAGCATGT | CATCGAGGCAGTTGTTCCCTT |
| *Cxcl12* | ATGGACGCCAAGGTCGTCGC | CGATGTGGCTCTCGAAGAAC |
| *Cxcr4* | TCAGTGGCTGACCTCCTCTT | CTTGGCCTCTGACTGTTGGT |
| *Egf* | GACTGAGTTGCCCTGACTCTAC | CACCACCATGATGTCATGCTTCTG |
| *Fgf2* | CTTGCTATGAAGGAAGATGGAC | CTGCCCAGTTCGTTTCAGTG |
| *Scf* | GTCCTAGAAGAAGCTTCTGGAGAG | GGTGGCTGAGTTGCTGACTC |
| *Ar* | GGGACCTTGGATGGAGAACT | GGTCTTCTGGGGTGGAAAGT |
| *Fshr* | AGCAAGGTGACCGAGATTCC | TCCCCAAATCCAGAAAATGA |
| *Esr1* | TCTCTGGGCGACATTCTTCT | CATGGTCATGGTAAGTGGCA |
| *Lhcgr* | TCCAGAGTTGTCAGGGTCG | TGGGATTACTTTGACAGGGA |
